# Let’s Be Clear—Health Impact Assessments or Assessing Health Impacts?

**DOI:** 10.3389/phrs.2024.1607722

**Published:** 2024-08-05

**Authors:** Jinhee Kim, Andrew Dannenberg, Fiona Haigh, Ben Harris-Roxas

**Affiliations:** ^1^Cities Institute, University of New South Wales, Sydney, NSW, Australia; ^2^ Department of Environmental and Occupational Health Sciences, School of Public Health, University of Washington, Seattle, WA, United States; ^3^ Department of Urban Design and Planning, College of Built Environments, University of Washington, Seattle, WA, United States; ^4^Centre for Primary Health Care and Equity, University of New South Wales, Sydney, NSW, Australia; ^5^ Sydney Local Health District, Sydney, NSW, Australia; ^6^School of Population Health, University of New South Wales, Sydney, NSW, Australia; ^7^School of Public Health, University of Technology Sydney, Sydney, NSW, Australia

**Keywords:** health impact assessment, health risk assessment, decision making, policy recommendations, stakeholder engagement

## Introduction

There has been a significant growth in health impact assessment (HIA)-related publications within peer-reviewed literature. These papers rely on authors’ accurate reporting of work as “health impact assessment” to contribute to the development of the field. In this commentary, we urge researchers to clearly differentiate between studies that “assess health impacts” and those that are specifically associated with the ex-ante tool formally defined as “health impact assessment,” which aims to support decision-making.

## Recent Growth of Scholarly Literature That “Assess Health Impacts”

In a recent article, Lamprecht et al. observed a significant increase in HIA scholarship over the past 15 years [1]. Most of the publications addressed what Lamprecht et al. categorise as “research-driven HIA.” HIA studies in this category “are not directly tied to decision-making processes of specific development initiatives, but instead are primarily driven by research interest” [1]. Lamprecht et al. note that the authors of these publications identify “health impact assessment” as a keyword to their work when they are in fact “impact evaluations” and are not designed as HIAs intended to support decision-making. Lamprecht et al. warn against the growing tendency to apply the term HIA to health impact modelling studies and quantitative health risk assessments.

We need to be clear: the term “research driven HIA” has no currency in the HIA field. Lamprecht et al. have used the term “research driven HIA” to describe impact evaluations and modelling studies, rather than suggesting that they are prospective assessments as understood within the field of HIA and across other forms of impact assessment, such as environmental impact assessment and social impact assessment. Since the earliest stages of its development as a distinct form of impact assessment, there has been a consensus that HIAs are intended to support decision-making and implementation prior to action, rather than being descriptions or evaluations of health impacts [2].

It has been widely recognized that HIAs should follow a systematic procedure encompassing screening, scoping, assessment, developing recommendations, reporting, and monitoring and evaluation [3]. The main findings of the HIA should include recommendations to the proposed policy, plan, project or program, aimed at maximizing the identified health benefits and mitigating the potential harms. Effective HIA requires active engagement with policy actors, stakeholders, and the impacted community to conduct the scoping phase, interpret the appraised health impacts, prioritize recommendations, and incorporate those recommendations into the final policy decisions [4].

Labelling all publications that estimate, model or describe health impacts as “research-driven HIA” is misleading. The use of this term not only contradicts Lamprecht et al.’s claim as being non-HIA [1], but contributes to the misuse of the term HIA in the literature. While the publications included in the systematic review were self-reported by their authors as HIA, HIA researchers should not inadvertently validate these types of research as HIA. For example, one can empirically appraise the past and current health impacts of climate change overall, but an HIA of the topic should focus on the potential health impacts of a specific proposed climate change policy and develop recommendations to inform decision-making [5].

Another problem with the label “research driven HIAs” is that it implies that “traditional” HIAs are not a form of research. Within HIA literature there has been debate about whether HIA is considered research [6]. Nevertheless, HIA actions align with the standards of research and scientific language. Data collection and analysis methods to find answers to the potential health impacts of the project, program or policy follow systematic and rigorous approaches [6].

## Advancing “Health Impact Assessment” Research in Scholarly Literature

A more pertinent discussion involves the under-representation of actual HIA studies in academic literature. The scholarly landscape of HIA-related studies repeatedly shows HIA case studies under-reported in academic literature despite its widespread application in jurisdictions globally [7]. While a simple explanation would cite publication bias and language bias, a more critical examination would involve the epistemological and methodological approaches favoured by the scientific community and its alignment with studies that assess health impacts. One reason few HIA reports are subsequently published may be that most HIA reports are written in a form and format that would need substantial revision to be suitable for academic publication and the report writers may have few resources and little motivation to do such revisions. While HIAs are generally conducted to influence specific decisions, they may or may not contain information that would be useful to a broader scientific community. To promote transparency and timeliness, final HIA reports should be posted on a publicly accessible website whether or not they are subsequently published. Examples of published HIAs of specific projects and policies include Hirono et al. [8] and Ross et al. [9].

In practice, HIAs are being more widely used in the private sector and as a requirement for internationally financed projects (e.g., World Bank, International Monetary Fund). More jurisdictions are institutionalising HIA as a mandatory policy requirement (e.g., European Union, Korea, Philippines, Wales). But because the primary purpose of these activities and their corresponding reports is intended to support decision-making, these reports are not typically published in the academic literature. In addition, mandated HIAs carried out as part of approval processes may not be publicly available and there is little incentive to publicise.

To advance the HIA scholarship, an international database of completed HIAs would add value to support future HIA practice and research. For example, the U.S. HIA database [10] and the now archived London HIA Gateway database, [11] have been valuable resources for HIA research [12].

Some areas of research that would be valuable to help advance HIA practice and research include:

• Better documenting impacts of HIA recommendations on subsequent health outcomes.
• Evaluating the impacts of HIAs conducted to meet World Bank, IMF and other international multilateral agency lending requirements on decision-making, implementation, and population health outcomes.
• Identifying cases where HIA has been routinely integrated into planning processes and its use sustained.
• Identifying methods for integrating HIA principles into routine public health, health management, urban planning, and transdisciplinary professional training.
• Working with private sector to identify and study HIAs conducted in that sector, which are otherwise not public processes and are poorly recognised in the literature.
• Identifying ways of strengthening/ensuring quality of HIAs within mandatory processes through, for example, research on quality assurance processes/standards.
• Enhancing methods for differentiating the distribution and characterisation of impacts within and between sub-populations and groups.

## Conclusion

Researchers should re-examine whether a study called an HIA is designed to support decision-making, or if it assesses health impacts but is unlikely to influence current decision-making. The latter studies should not be called HIAs, but might be labelled as impact evaluation, assessing health impacts, or modelling health impacts.

Considering the decision support objective of HIA, peer-reviewed academic literature is not the most appropriate platform to access publications on HIA-related cases and studies because most HIAs are never published. Greater efforts to sustain and enhance international HIA repositories are needed. We also urge the HIA community to support HIA-related academic publications by developing reporting guidelines for academic HIA literature and accelerating personal and institutional efforts to report learnings from HIA cases in peer-reviewed scholarship.
